# Permeability modelling in a highly heterogeneous tight carbonate reservoir using comparative evaluating learning-based and fitting-based approaches

**DOI:** 10.1038/s41598-024-60995-7

**Published:** 2024-05-03

**Authors:** Ehsan Hajibolouri, Ali Akbar Roozshenas, Rohaldin Miri, Aboozar Soleymanzadeh, Shahin Kord, Ali Shafiei

**Affiliations:** 1 Petroleum Engineering Program, School of Mining & Geosciences, Nazarbayev University, 010000 Astana, Kazakhstan; 2 School of Chemical Engineering, Iran University of Science and Technology (IUST), PO Box 16765-163, Tehran, Iran; 3 Department of Geosciences, University of Oslo, Blindern, PO Box 1047, 0316 Oslo, Norway; 4 Department of Petroleum Engineering, Ahwaz Faculty of Petroleum, Petroleum University of Technology, Ahvaz, Iran

**Keywords:** Machine learning, Random forest, Permeability modelling, Heterogeneity, Reservoir simulation, Data modelling, Core processes, Geodynamics

## Abstract

Permeability modelling is considered a complex task in reservoir characterization and a key component of reservoir simulation. A common method for permeability modelling involves performing static rock typing (SRT) using routine core analysis data and developing simple fitting-based mathematical relations that link permeability to reservoir rock porosity. In the case of carbonate reservoirs, which are associated with high heterogeneities, fitting-based approaches may fail due to porosity–permeability data scattering. Accurate modelling of permeability using petrophysical well log data seems more promising since they comprise a vast array of information about the intrinsic properties of the geological formations. Furthermore, well log data exhibit continuity throughout the entire reservoir interval, whereas core data are discrete and limited in availability and coverage. In this research work, porosity, permeability and log data of two oil wells from a tight carbonate reservoir were used to predict permeability at un-cored intervals. Machine learning (ML) and fitting models were used to develop predictive models. Then, the developed ML models were compared to exponential and statistical fitting modelling approaches. The integrated ML permeability model based on Random Forest method performed significantly superior to exponential and statistical fitting-based methods. Accordingly, for horizontal and vertical permeability of test samples, the Root Mean Squared Error (RMSE) values were 3.7 and 4.5 for well 2, and 1.7 and 0.86 for well 4, respectively. Hence, using log data, permeability modelling was improved as it incorporates more comprehensive reservoir rock physics. The outcomes of this reach work can be used to improve the distribution of both horizontal and vertical permeability in the 3D model for future dynamic reservoir simulations in such a complex and heterogeneous reservoir system.

## Introduction

Permeability of reservoir rocks is typically measured using a limited number of core samples taken from wells. Advances in technology such as Nuclear Magnetic Resonance (NMR) log have made it possible to estimate permeability with a high accuracy using in situ measurements in an interval, continuously. However, permeability distributions between wells remains a significant challenge in formation evaluation. Various algorithms and mathematical methods are available for prediction of permeability in un-cored intervals, known as permeability modelling. This is crucial for preparing flow simulation models to evaluate reservoir performance. 3D dynamic reservoir models are essential for field development plans, reservoir performance prediction, well-based operations, and enhanced oil recovery (EOR) screening. Prediction of permeability throughout un-cored intervals is a complex process and researchers have attempted to develop a universal correlation covering these complications^1–4^. Machine learning (ML) is a powerful tool with a wide range of rapidly growing applications, which can be trained with well-based and reservoir-based data for permeability modelling purposes. Several techniques are available for permeability measurement including core scale, reservoir scale, testing, and NMR logs^5^. In routine core analysis (RCAL), permeability is measured on core scale; while, in transient well testing it is measured on a large volume of reservoir rock, reducing the uncertainty. Formation testing reports permeability of a specific spot in the well column, resulting in limited heterogeneity ranges compared to transient testing^6^. However, different length scales and heterogeneity scales result in different permeability values.

Permeability modelling techniques can be classified into three general groups: (1) Mathematical modelling based on measurable rock properties (e.g., porosity, grain size, and tortuosity) such as the exponential Kozeny–Carman relation^7,8^, correlations based on irreducible saturation^9,10^, and permeability model by Pittman and Winland based on porosity and pore diameter^11,12^, (2) Statistical permeability models such as Pearson-Tukey and Swanson averaging^13,14^, and (3) intelligent techniques such as ML and Artificial Intelligence (AI) modelling^15,16^. Empirical permeability models have experienced a significant progress in recent years thanks to availability of various methods to assess gas permeability and diffusivity in tight reservoirs. The tools used for this purposed include numerical models, NMR measurements, and fractal theory-based approaches to better understand gas transport properties in complex geological formations^17–20^. Correlations developed for specific reservoir and petrology conditions, even in similar geological and petrological conditions, are rarely successful in different reservoirs. Hence, some parameters related to rock physics are necessary to determine the governing rules between measured/available data and rock permeability. Log data are the most potential suitable candidates for this application. They have attracted growing attention because of: (i) their abundance and availability, (ii) good areal distribution and vertical continuity through geological structure of the reservoir, and (iii) each log parameter represents specific physics of the reservoir rock (such as sonic characteristics, natural or radioactivity, and electric features). Implementing log data (such as sonic, gamma, resistivity, neutron, density, and NMR) instead of macro/microscopic features of reservoir rock (e.g., porosity, specific surface area, tortuosity, and pore size) in permeability modelling has attracted much attention recently. Among the log parameters, electrical resistivity and sound speed are the most correlated with rock permeability since they are affected by rock permeability^21^. Furthermore, permeability of carbonate reservoirs can be estimated using NMR logs, accurately^22^. However, NMR data are not always available compared to routine full-set log parameters. Thus, some alternative methods should be developed for permeability modelling based on conventional log data. Because the measurement of pore size, grain radius, tortuosity, and irreducible water saturation is challenging in routine permeability modelling methods, some researches assessed these shortcomings^5^. Other researchers used log data with multi-parameter regression techniques^5,9^. However, they did not perform well in permeability modelling of heterogeneous sandstones and carbonate reservoirs^15^. Hence, researchers recently used artificial neural networks to obtain more accurate and acceptable results^15,23^.

Intelligent methods and, more specifically, ML methods have been used frequently for various engineering purposes. When the objective variable is a constant value (not distinct classes or categories), regression ML methods must be used. Hence, the problem of permeability modelling is categorized as a regression problem. Since there is a remarkable (vertical and areal) variety in correlation between permeability (*k*) and porosity (Φ), a single correlation cannot lead to accurate permeability predictions in all cells of a reservoir model. ML methods are able to resolve this position dependency of *k*-Φ relations^24^. Several pieces of research used ML methods in the permeability modelling of petroleum reservoirs^25–51^. A summary of application of some AI models for reservoir properties prediction is presented in Table 1.

**Table 1:** Application of some AI models used previously in reservoir properties prediction.

Author(s)	Applied algorithm	Input parameters	Prediction target	Performance
^31^	FL	GR, core porosity	*k*	R = 0.98
^32^	SVM	GR, NPHI, DT, DEN	Φ	0.5 < R < 0.8
^33^	SVM	GR, NPHI, DT, DEN, RT	*k*	0.4 < R < 0.82
^34^	SVM	RHOB, LLD, LLS, DT, MSFL, GR, NPHI, Coordinates X and Y	*k*	R = 0.96 MSE = 0.07
	GRNN			R = 0.94 RMSE = 0.12
^35^	Functional networks	CT, DEN, DT, MSFL, NPHI, PHIT, RT, SWT	*k*	R^2^ = 0.93
^36^	ANN	GR, RD, DEN, NPHI, PHID	Φ	R = 0.99
			*k*	R = 0.99
^37^	GA-FL, GA-LSSVM	DT, DEN, CNL, PHIT	Φ	R^2^ = 0.97
			*k*	R^2^ = 0.99
^38^	ANN	NPHI, RHOB, DT, GR, RL	*k*	R = 0.82 MSE = N/A
^39^	ANN-ICA	NPHI, RHOB, DT, log(RT), log(*k*)	Φ	R^2^ = 0.91
			*k*	R^2^ = 0.89
^40^	ANN	RHOB, NPHI, PHIE, LLD, SFL, DT	*k*	R = 0.94 ARE = 0.01
^41^	ANFIS	NPHI, RHOB, PEF, GR PHIE, LLD, S_w_, GR	Φ	R = 0.99 NMSE = 2.07 × 10^–4^
			*k*	R = 0.99 NMSE = 8.64 × 10^–5^
^42^	ANN	RL, NPHI, RHOB	*k*	RMSE = 0.28 R = 0.95
^43^	ICA-ANN	RHOB, NPHI, PHIT, DT	Φ	R = 0.90 MSE = 1.41 R = 0.92 MSE = 0.96
			*k*	
	HGAPSO-ANN		Φ	R = 0.98 MSE = 0.39 R = 0.98 MSE = 1.41
			*k*	
	HGAPSOLSSVM		Φ	R = 0.98 MSE = 0.96 R = 0.99 MSE = 0.39
			*k*	
^44^	CNN	GR, DEN, the slopes of the GR and DEN curves, VSH	*k*	R^2^ = 0.92
^45^	PSO-MKF-SVM	GR, DEN, the slopes of the GR and DEN curves, VSH	Φ	R^2^ = 0.91
^46^	ANN, GA	Φ, pore throat diameter—log(d_pt_), log(F)	*k*	R^2^ = 0.38 R^2^ = 0.43
^47^	GMDH	RHOZ, RLA5, GR, RLA1, TNPH, VSH	*k*	R = 0.86
^48^	PSO-SVM	GR, DEN, DT, their slopes	*k*	R^2^ = 0.83
^49^	DBS-Decision Tree	Micro-CT images of estaillades limestone	*k*	RMSE = 0.43
^50^	RF	Grain density, Φ, *k*	*k*	R^2^ = 0.83
^51^	GMDH-LM	GR, RHOZ, PHIE, VSH, TNPH	*k*	RMSE = 0.67 MAE = 0.05
	Conventional GMDH			RMSE = 0.69 MAE = 0.04
	BPNN			RMSE = 0.75 MAE = 0.02

According to the literature, several research work have been performed on intelligent permeability modelling. However, few researches addressed the application and comprehensive comparison between fitting-based and learning-based permeability modelling methods. In the petroleum industry, permeability is usually predicted using fitting-based approaches. The prediction of permeability can be improved by applying learning-based techniques; particularly when more log data from the reservoir is included following well drilling. These techniques are more economical since they exhibit higher levels of completeness and experience fewer errors. While, in this research work, we practically demonstrated the ability of ML methods to effectively handle the disparities and uncertainties inherent in the input data, thereby yielding more reliable and promising predictions of permeability. Moreover, this research work is performed on a challenging database that belongs to a highly heterogeneous, low porosity/permeability, and tight oil reservoir with average porosity and permeability of 0.05 and 2 mD, respectively.

In this paper, a brief description of the basics of exponential, statistical, and ML permeability modelling methods is provided (“Permeability modelling methods” section) followed by description geological setting, core and log data of an Iranian tight carbonate oil reservoir. Then, the fitting-based and learning-based permeability modelling methods were applied to the database. The efficiency of permeability prediction is then assessed and compared for all models. In the final stage, permeability modelling is performed and its reliability is verified via comparison with the average values of core permeability in all of the wells.

## Permeability modelling methods

### Geological setting and database

**Figure 1:**
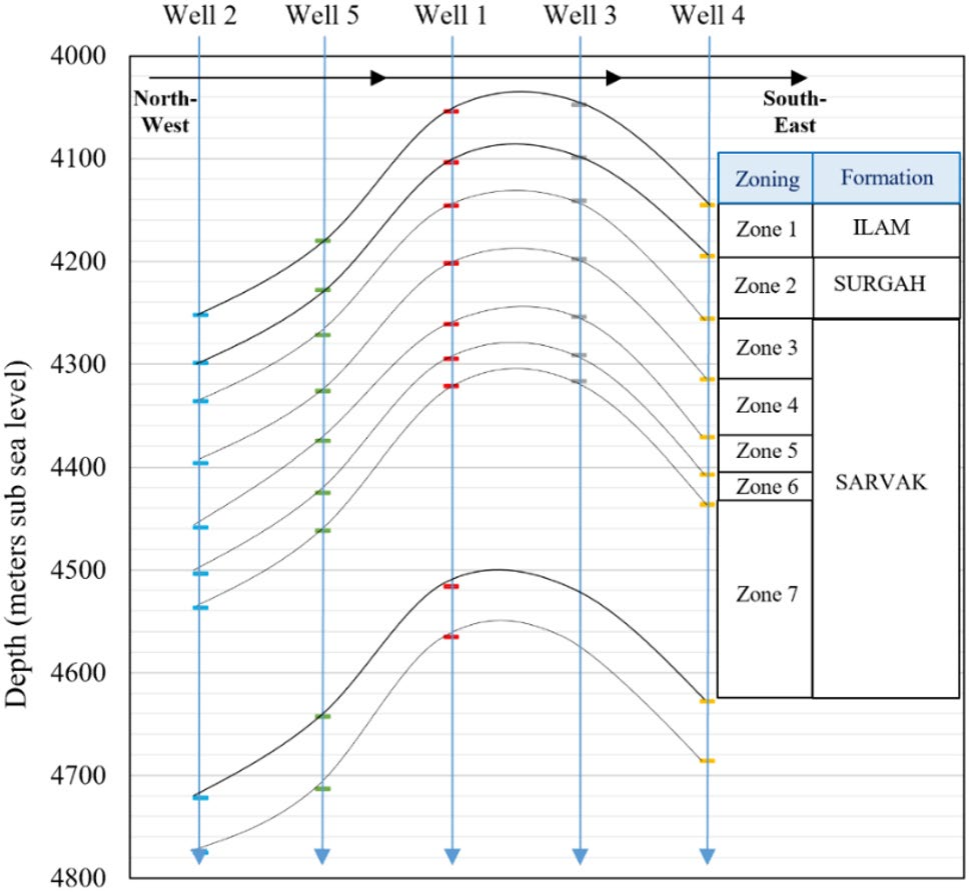
A schematic view of relative well positions, depth of formations tops, and the zones crossed by wells 1–5.

This research work is performed on one of the Iranian south oil reservoirs trapped in a structural anticline. The study area contains three formations: Ilam, Surgah and Sarvak, and the main reservoir in the field lies in the Sarvak formation, with primary lithology of calcite and dolomite and thin layers of shale. The reservoir can be categorized as a tight carbonate reservoir with an average porosity of 0.021 and water saturation of 0.64. Moreover, according to facies analysis, the main reservoir is composed of low to medium quality carbonate rocks and only about 10% of the reservoir rock can be characterized as potential reservoir rocks. The main oil-bearing layers are zones 4 and 6 (part of the Sarvak formation), compact carbonate formations with low porosity/permeability characteristics. A schematic of relative well locations, geological zoning, and the crest and lateral wells are presented in Fig. 1.

Five wells were drilled and completed in this reservoir including four production wells and one observation well. Due to the limited number of wells, close distance, and low distribution of wells through the reservoir geological structure, the characterization of reservoir rock, and permeability modelling are quite challenging. Petrophysical log data are available for all five wells; however, laboratory porosity/permeability core data are only available for wells 2 and 4. The lack of experimental data and the fact that the wells number 2 (northwest) and 4 (southeast) are located in the two furthest locations of the reservoir (about 10 km distance) are two factors that add to the level of uncertainty and challenge of the present research work.

Here, we first provided a statistical view of the experimental data because statistical parameters represent a comprehensive view of the dataset and the reservoir characteristics. For example, average values indicate the range of expected values from permeability modelling, and standard deviation reveals the dispersion and distribution of the data around the mean value. A detailed statistical description of the porosity/permeability data of wells 2 and 4 is presented in Table 2. The average permeability of the wells 2 and 4 is 1.86 and 1.15 mD for horizontal, and 2.29 and 0.88 mD for vertical samples. Additionally, most of the core samples in these two wells belong to the zones 4 and 6, the potential oil-bearing layers. Hence, the final permeability model is expected to show reliable performances in the oil-bearing zones.

**Table 2:** Statistical parameters (number of cores, average and standard deviation of permeability, and average porosity) for *k*-Φ core samples in all zones of the wells 1 and 2.

		Horizontal samples				Vertical samples			
		Number of samples	Average *k* (mD)	STD of *k*	Average Φ (%)	Number of samples	Average *k* (mD)	STD of *k*	Average Φ (%)
Well 2	Zone 4	110	3.34	5.88	6.36	61	3.79	6.96	5.05
	Zone 5	94	1.56	5.12	2.76	51	0.25	1.16	5.18
	Zone 6	58	2.22	4.90	8.38	29	2.74	6.70	9.99
	Overall	262	2.58	5.46	6.02	141	2.29	5.72	5.27
Well 4	Zone 4	57	1.85	5.42	2.83	40	0.47	0.57	3.17
	Zone 5	–	–	–	–	–	–	–	–
	Zone 6	50	1.10	1.01	9.30	20	1.98	2.39	9.55
	Zone 7	16	0.15	0.12	1.69	7	0.08	0.041	1.10
	Zone 8	15	1.02	1.92	4.31	10	1.98	3.58	4.47
	Zone 9	8	0.91	1.08	3.43	6	0.21	0.18	2.90
	Zone 10	20	0.25	0.22	5.10	11	0.21	0.27	5.40
	Overall	166	1.15	3.32	5.11	94	0.88	1.77	4.76

**Figure 2:**
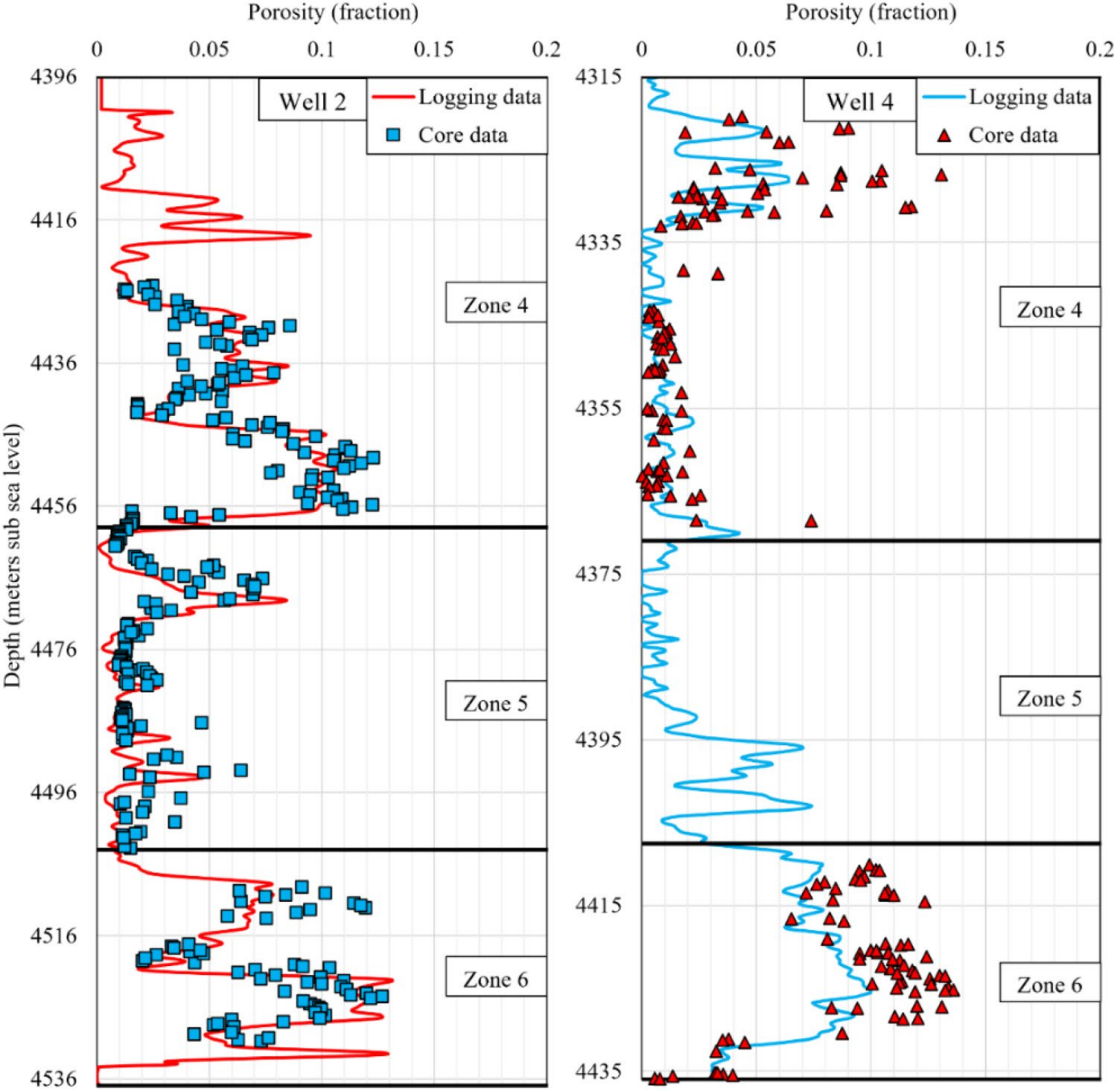
Matching log porosity with core porosity versus depth for the zones 4, 5 and 6 in the wells 2 and 4, where core samples are available.

Log and core porosity data of the zones 4, 5, and 6 in the wells 2 and 4 are presented in Fig. 2. There is an acceptable match between the laboratory and log porosity data in the well 2. However, in some intervals of the well 4, there are discrepancies originating from previous data acquisition processes. In the upper parts of the zone 4, log porosity is half of the value reported by core samples, and in the zone 6, log porosity is about 2/3 of the core porosity values. The porosity data does not have the same quality in all the wells and zones, so the proper modelling method should mitigate this inconsistency, reducing the level of uncertainty in permeability prediction. Furthermore, because of the poor quality of the porosity data and the tight and heterogeneous nature of the reservoir rock, conventional fitting-based methods are not deemed viable options as they typically rely on porosity data for permeability predictions. Hence, use of other available data (i.e., log data) in permeability prediction alleviates the negative impacts of data discrepancy and helps to manage the high uncertainty present in the dataset.

A complete set of log data is available for all of the five wells. The log dataset includes various data columns such as bit size (BS), Caliper (CALI), total conductivity (CT), sonic (DT), neutron porosity (NPHI), density (RHOB), total resistivity (RT), spectral gamma ray (SGR), calcite volume (VOL_CALCITE), dolomite volume (VOL_DOLOM), oil volume (VOL_UOIL) and water volume (VOL_UWATER). The availability of log data columns in each well is presented in Table 3. The available data are denoted in green and the missing data in red. A general permeability model must be developed based on the data columns available in all of the wells. Hence, eight data columns (known in all of the five wells) having physical relations with the pore geometry of the reservoir rock were chosen as input variables for the ML modelling conducted in this research work.

**Table 3:**
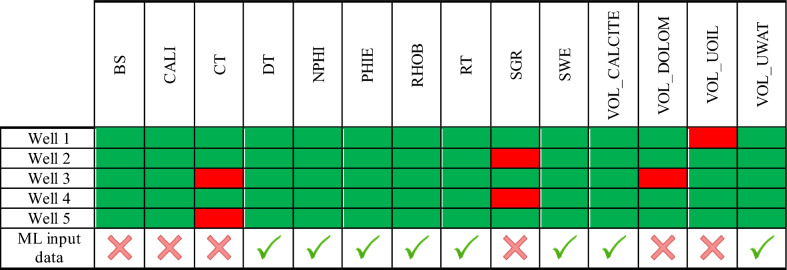
Availability of log data in wells 1 to 5, and log parameters used for ML modelling.

### Reservoir rock typing

Rock typing can be defined as dividing reservoir rock into distinct categories, each having a specific condition of geological deposition and diagenetic features^52^. Successful rock typing leads to a reliable permeability prediction, saturation height modelling, dynamic simulations, and performance forecasting^53^. Several rock typing methods have been introduced in the literature, each of which is developed for specific lithology, rock quality, and geological conditions^52–54^. In this research work, we used the most popular methods (Rock Quality Index (RQI), Flow Zone Indicator (FZI), Discrete Rock Typing (DRT), and Winland methods) on the vertical and horizontal core *k*-Φ data as a part of the fitting-based methods. FZI method showed the best performance in classifying the data into distinct rock types. Since the main objective of this research work was to compare fitting-based and learning-based permeability modelling approaches, and to avoid prolongation of the paper, only the most widely used rock typing method (the FZI method) are presented here. To apply the FZI method, the RQI is first calculated as:

1 $$RQI=0.0314\times \sqrt{\frac{k}{{\Phi }_{e}}}$$

where *k* is the sample permeability in mD, ${\Phi }_{e}$ is the effective porosity, and RQI is in μm. FZI is defined as the ratio of RQI to normalized porosity (${\Phi }_{z}$) as:

2 $$FZI=\frac{RQI}{{\Phi \Phi }_{z}}$$

3 $${\Phi }_{z}=\frac{{\Phi }_{e}}{1-{\Phi }_{e}}$$

where FZI is in $\mu m$. Distinct rock types can be identified by: (i) taking core samples with similar FZI values as a distinct rock type or (ii) plotting RQI against ${\Phi }_{z}$ in log–log scale, then each unit slope line represents a rock type.

### Statistical correlations

Delfiner^21^ performed a statistical investigation on *k*-Φ relations and pointed out that rock typing methods based on exponential *k*-Φ relations lead to underestimation of permeability. These methods result in arithmetic permeability averages lower than laboratory average values. Hence, these method would lead to underestimated values in the 3D upscaled model of a reservoir^21^. As Male and Duncan showed, exponential relations may lead to underestimation of core permeability by up to 3 times or more, particularly in heterogeneous carbonates^55^. To prevent this modelling bias, a statistical averaging method based on Swanson averaging was applied to core data to develop a *k*-Φ relation having a lower prediction bias and closer arithmetic average^21^. Firstly, the total porosity interval is divided into some sub-intervals, then 10%, 50%, and 90% quintiles of each sub-interval are calculated, and finally, the Swanson average of each is calculated as follows:

4 $${X}_{mean}=0.3{X}_{10}+0.4{X}_{50}+0.3{X}_{90}$$

where ${X}_{10}$, ${X}_{50}$, and ${X}_{90}$ are 10%, 50%, and 90% quintiles, respectively. After ${X}_{mean}$ values are calculated, and a correlation is fitted for ${X}_{mean}$ against Φ, and it is used for permeability modelling instead of exponential *k*-Φ relations.

### Machine learning permeability modelling

**Figure 3:**
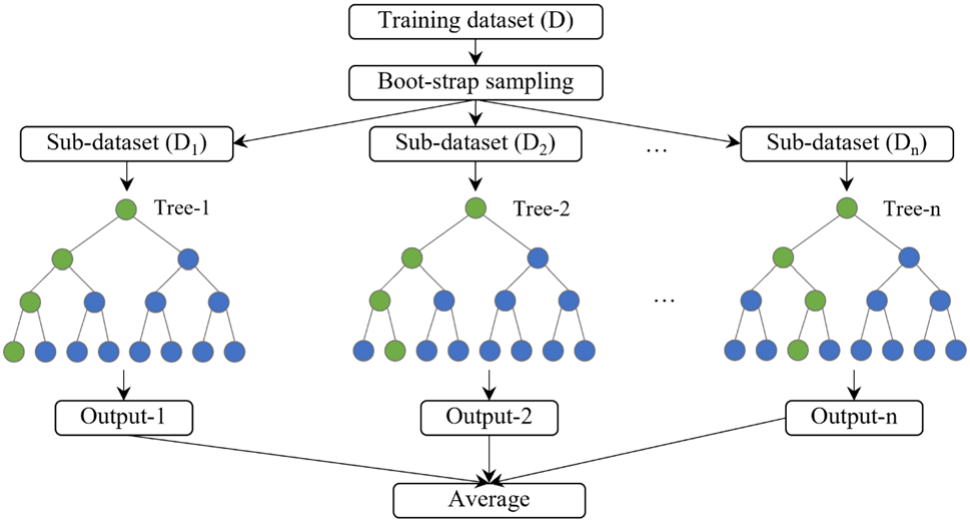
Workflow of the random forest algorithm^56^.

In recent years, ML methods have been widely used for reservoir property modelling. There are several commonly used ML methods such as ANN, SVM, Gradient Boosting Regressor (GBR), Lasso Regressor, K-nearest Neighbors (KNN), Decision Tree (DT), and Random Forest (RF) algorithms. Since model selection is an essential step in ML predictive modelling, multiple algorithms were trained on the same dataset to compare their performances in both the training and validation steps. For the present dataset, the RF Regressor was found as the most suitable ML model (see “Learning-based methods” section). The model selection process should be repeated for each database indicating the random forest model is not a universal method that performs best for all databases. As several models are used to maximize the performance, it is not feasible to describe in detail the design of each model within the scope of this research work. Here, we focused on explaining the Random Forest structure, a key model in our work. Hence, this algorithm is briefly discussed here. The RF is a robust integrated ML algorithm developed by Breiman^56^. The primary aim of developing the RF algorithm was to solve unsupervised regression and classification problems. However, it has been successfully applied to supervised regression problems such as porosity prediction from wireline log data^57^. In other words, the main concept behind this technique was building several independent decision trees also known as ensemble of trees and training them on the desired dataset and finally to make predictions. This algorithm utilizes bootstrap resampling technique to avoid overfitting, a resampling approach which works via replacement. Bootstrap sets are then formed from initial data where several samples are replaced with other repeating samples. Each tree is then built on an individual bootstrap set in the RF algorithm. Hence, all the trees would be different as they were built on diverse datasets leading to unalike predictions. In the next stage, all the trees are aggregated together, and the final prediction is obtained by averaging the predictions of individual trees (in the case of regression)^56–58^. The RF method can provide the importance degree of each feature and pairwise proximity between samples. Conversely, the RF algorithm favours smaller groups over larger groups if the input data contains groups of correlated features of similar relevance. The workflow of the random forest algorithm is presented in Fig. 3.

## Results and discussion

### Fitting-based methods

#### Rock typing permeability modelling

**Figure 4:**
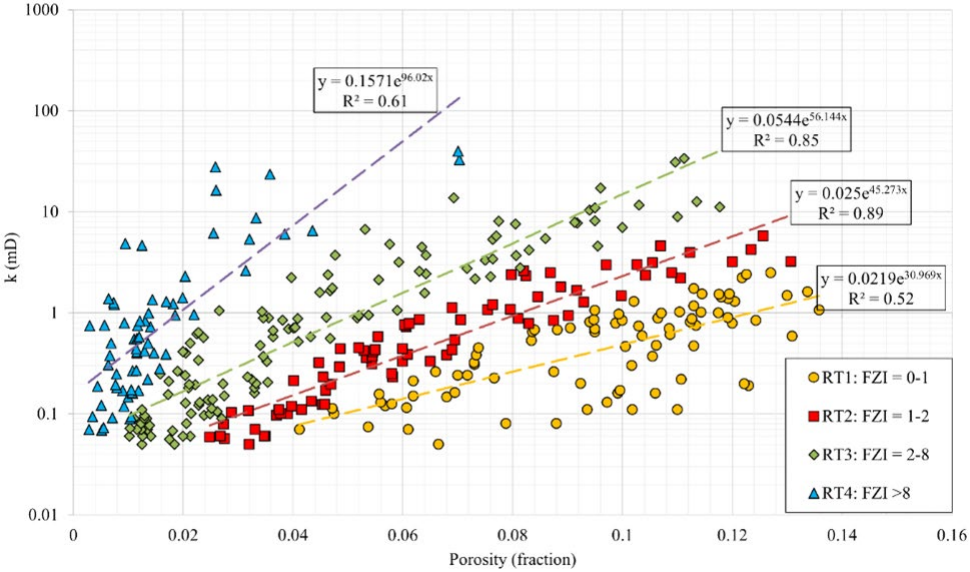
Horizontal core permeability versus porosity, FZI rock types and rock typing equations.

**Figure 5:**
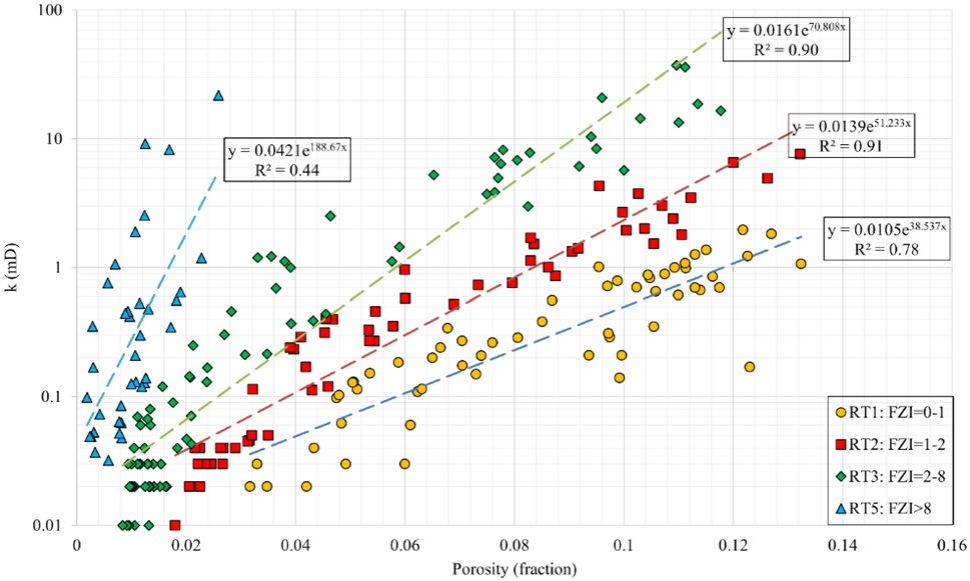
Vertical core permeability versus porosity, FZI rock types, and rock typing equations.

In this section, the FZI rock typing and permeability modelling is described as it performed the best in classifying the data into distinct rock types (RTs) among various methods used. As shown in Fig. 4, horizontal permeability was classified into four RTs (distinct FZI intervals). Exponential *k*-Φ correlations were obtained for the RT1 to RT4 with relatively good determination coefficients of 0.68, 0.89, 0.84, and 0.61. The RT1 and RT4 showed weaker correlations because they included the data points with scattered FZI values. This reveals that as the values of FZI and average permeability increases, the average porosity and storage capacity of reservoir rock decreases by a factor of 5.7, from the RT1 to RT4. This procedure was also repeated for vertical data resulting in four RTs with the same FZI intervals as in the horizontal samples. The data points and exponentially fitted correlations are presented in Fig. 5. The RT1 to RT3 were fitted with proper correlation coefficients of 0.782, 0.908, and 0.902, respectively. According to the statistical analysis, as the average vertical permeability increases, the average porosity decreases from the RT1 to RT3. The RT4 showed a very weak correlation coefficient because (i) its data were highly scattered, and (ii) while the average permeability of the RT4 is roughly 1/3 of the RT3, the lower porosity of RT4 resulted in higher FZI values. This demonstrates the incompetency of this permeability modelling method.

The permeability model obtained by rock typing is not applicable in un-cored reservoir depths because porosity and fluid saturations are the only available data in these locations, and they are not enough to identify the RT of each un-cored location. A simple and imprecise method is to take all permeability samples as a single rock type to achieve a general *k*-Φ relation, which can be used for permeability modelling of un-cored regions.

#### Statistical permeability modelling

**Figure 6:**
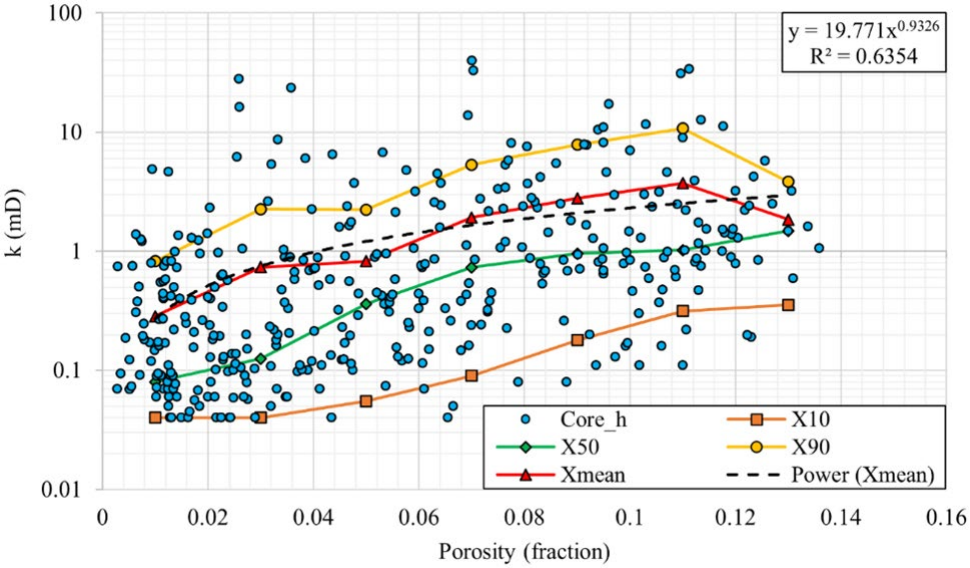
Horizontal core permeability versus porosity for statistical permeability modelling, and the final rock typing equation.

**Figure 7:**
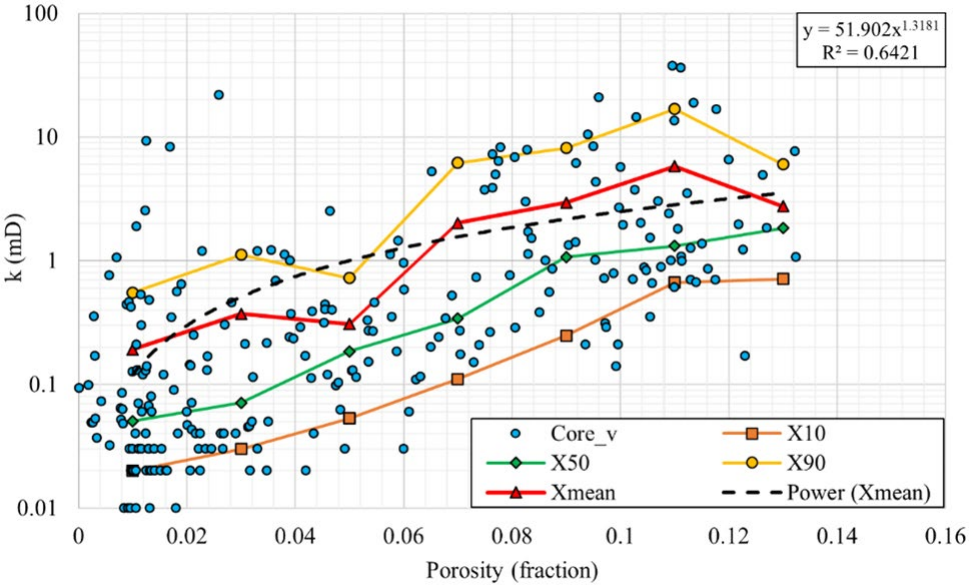
Vertical core permeability versus porosity for statistical permeability modelling, and the final rock typing equation.

A statistical permeability modelling method introduced by Delfiner^21^ was also applied to the data to compare the permeability modelling methods, comprehensively. This approach is expected to reduce the pessimistic effects of the exponential fitting on average predicted permeability values which can led to underestimation of the core permeability values by a factor of 3^55^. In this section, statistical permeability modelling for horizontal permeability data (Fig. 6) and vertical permeability data (Fig. 7) are presented and discussed. For a detailed description of the applied procedure, one can refer to the paper by Delfiner^21^.

The values of ${X}_{10}$, ${X}_{50}$ and ${X}_{90}$ were calculated and plotted for each 0.02 porosity interval. Afterward, the Swanson averaging method (Eq. 4) was used to calculate the average values (${X}_{mean}$). Then, power correlations were fitted to the average values to obtain the final permeability modelling correlations, which showed acceptable coefficients of determination (R^2^) of 0.87 and 0.77 for horizontal and vertical permeability models. Since the R^2^ does not represent the efficiency of permeability modelling, comprehensively, the proficiency of this method is evaluated and compared with other methods in Sect. 5.

### Learning-based methods

The choice of machine learning (ML) model within a pool of models should align with the characteristics of the dataset. It is important to acknowledge that no model is flawless. Owing to both intrinsic limitations within the dataset and inherent imperfections in all models, each carrying its own set of limitations and degrees of error. The effectiveness of a model is determined by how well it meets the predefined criteria. Upon identification of the most suitable model, a subsequent step involves optimization and evaluation of the model to enhance its efficiency when applied to a given dataset.

**Figure 8:**
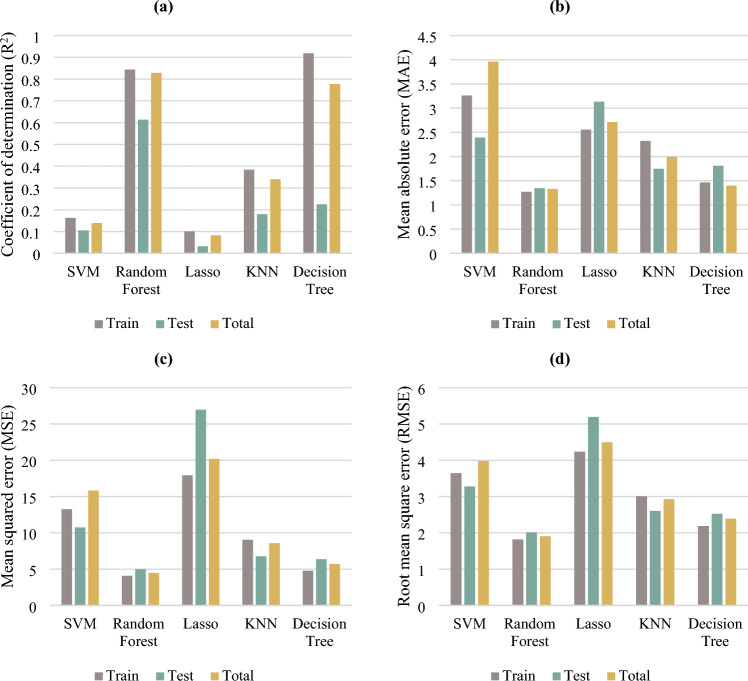
(**a**) R^2^, (**b**) MAE, (**c**) MSE, and (**d**) RMSE for the training, testing, and the overall dataset for SVM, RF, Lasso, KNN and DT machine learning methods in ML model selection process.

In this research work, the following AI algorithms were examined: SVM, RF, Lasso regression, KNN, and DT. Then, four separate groups of training/testing data with a ratio of 4:1 (train: test) were created and each algorithm was applied to all of the four datasets. The performance of the models then was assessed using different statistical quality measures including the R^2^ (Eq. 5), mean absolute error (MAE) (Eq. 6), Mean Squared Error (MSE) (Eq. 7), and Root mean squared error (RMSE) (Eq. 8) values, which are calculated and reported for train, test, and overall data sets (see Fig. 8) to compare and select the most effective algorithm.

5 $${R}^{2}=1-\frac{{{({Actual}_{i}-Predicted}_{i})}^{2}}{{({Actual}_{i}-mean \; of \; the \; observed \; data)}^{2}}$$

6 $$MAE=\frac{\sum_{i=1}^{N}\left|{Actual}_{i}-{Predicted}_{i}\right|}{N}$$

7 $$MSE=\frac{\sum_{i=1}^{N}{{({Actual}_{i}-Predicted}_{i})}^{2}}{N}$$

8 $$RMSE=\sqrt{\frac{\sum_{i=1}^{N}{{({Actual}_{i}-Predicted}_{i})}^{2}}{N}}$$

where, N is the total number of observations.

As shown in Fig. 8, the Lasso regression and SVM algorithms demonstrated the weakest performance on the present dataset. The KNN model has shown a weak to intermediate prediction such that the training and testing R^2^ were 0.38 and 0.18. The critical point is that the decision tree model is mostly over-fitted, evidenced by the high training R^2^ of 0.92 and the low test R^2^ of 0.22. As a result, the RF model retained a relatively reasonable accuracy in permeability prediction from log parameters with an average R^2^ values of 0.84 (train) and 0.62 (test), MSE values of 4.0879 (train) and 4.989 (test), RMSE values of 1.819 (train) and 2.01 (test), and MAE values of 1.19 (train) and 1.34 (test) for the respective datasets.

Although the accuracy of the ML permeability modelling is not excellent because of the scattered and highly heterogeneous nature of the present dataset, this method is more precise and comprehensive than fitting-based methods, which only fit a few equations to all the data points, even in high heterogeneity situations. Besides, the following advantages can be listed for ML permeability modelling in comparison to fitting-based methods:

i. The ML models are more comprehensive than fitting-based models because they predict rock permeability based on multiple (log) parameters rather than just using porosity and the fact that the log data are more abundant and available than core and laboratory data.
ii. The ML models operate based on learning the rules between the input and objective parameters; while, the fitting-based methods use only some porosity/permeability correlations.
iii. The ML models sufficiently account for the areal and vertical heterogeneity of the reservoir because log data is usually available both areally and vertically.
iv. Each log parameter represents specific physics from the porous media (sonic, electrical, neutron, and radioactive), which strengthens the physical basis of this approach instead of only using porosity values.

After the best model is selected, it must be trained on the present dataset using an appropriate strategy. In this step, the main question is how to feed the input data into the models for the training process? Considering the available permeability (training) data, the following potential structures are suggested for the training process. (i) Well-based model: train the models and perform predictions for four groups: horizontal data of well 2, vertical data of well 2, horizontal data of well 4, and vertical data of well 2, separately. (ii) Integrated model: Develop two general models by integrating all horizontal samples for one model and all vertical samples for another.

Depending on the expected results and applications of the permeability modelling, one of the mentioned approaches should be followed for training and prediction. For instance, when there are suitable areal and vertical distributions of wells with available core permeability and log data, the well-based approach is more accurate but time-consuming. On the other hand, when there are good distributions of wells and log data, but core permeability data only exist for a few wells, the integrated approach is the best choice. In the following sections, we presented the results of both methods to provide a comprehensive comparison. In this research work, the integrated approach is preferable because:

i. As mentioned in “Results and discussion” section, log data are available from all the wells (wells 1 to 5). However, core data is only accessible from wells 2 and 4.
ii. Integrated modelling approach provides two general models (horizontal and vertical), which can model rock permeability wherever log data are available. This method results in 10 permeability logs for wells 1 to 5 (five horizontal and five vertical), increasing the availability of areal and vertical permeability data and consequently enhancing the efficiency of geo-statistical property distribution in the 3D reservoir model.
iii. From the perspective of ML modelling, as the database size increases, the obtained model shows a higher accuracy and reliability in predictions. Herein, the integrated ML models are trained with a database much more extensive than in well-based methods. Hence, the results of the integrated ML modelling are more reliable.

In this section, we applied the well-based approach to the dataset, and four RF models were trained and tested for permeability predictions, separately. The tuned and optimized hyper-parameters for each model are presented in Table 4. The models were trained and tested with relatively good quality as evidenced by a R^2^ of 0.80. Then, the vertical and horizontal permeability of the wells 2 and 4 were predicted. The performance of permeability prediction using this approach is examined in detail and compared with other methods in Sect. 5.

**Table 4:** Hyper-parameters and R-squared of RF model trained in each part of the present research work (well-based and integrated approaches).

Modelling approach	Model	Samples count	Bootstrap	Criterion	max_depth	max_features	max_leaf_nodes	n_estimators	min_samples_leaf	min_samples_split	R^2^ train	R^2^ test
Well-based ML	Well 2_h	262	True	Squared_error	17	8	20	500	1	2	0.82	0.83
	Well 2_v	141	True	Squared_error	9	2	18	100	1	2	0.87	0.82
	Well 4_h	166	True	Squared_error	8	7	23	1000	2	2	0.81	0.80
	Well 4_v	94	True	Squared_error	20	8	18	500	1	2	0.80	0.78
Integrated ML	Horizontal	428	True	Squared_error	10	8	30	50	1	2	0.84	0.76
	Vertical	235	True	Squared_error	15	8	30	300	1	2	0.85	0.82

To apply the integrated approach, two distinct databases were established: one for total horizontal samples and another for total vertical core data of the wells 2 and 4. Then, the hyper-parameters of RF models were tunned, and the models were trained and tested. The test and train determination coefficients of 0.84 and 0.76 for horizontal, and 0.85 and 0.82 for vertical samples (see Table 4) demonstrate the high accuracy of the integrated models. Although a high determination coefficients is a good characteristic for a regression ML model, but it is not comprehensive and does not guarantee successful predictions. Hence, a detailed and comparative performance analysis of all the applied methods is presented in Fig. 8.

**Figure 9:**
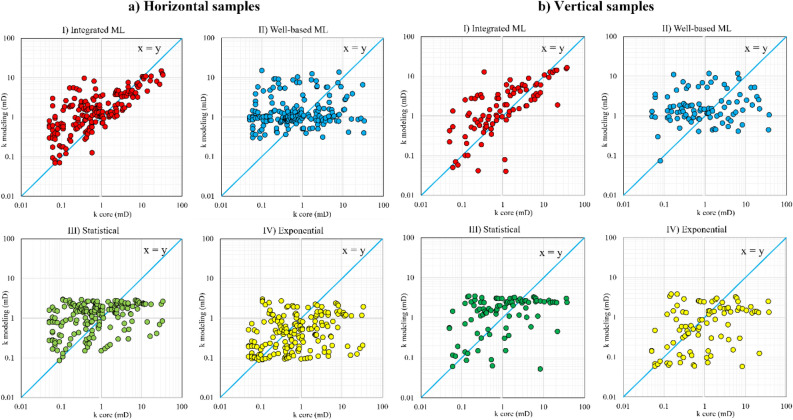
Modelling permeability versus core permeability for (**a**) horizontal core samples and (**b**) vertical core samples from the well 2; (I) integrated ML, (II) well-based ML, (III) statistical, and (IV) exponential fitting models.

**Figure 10:**
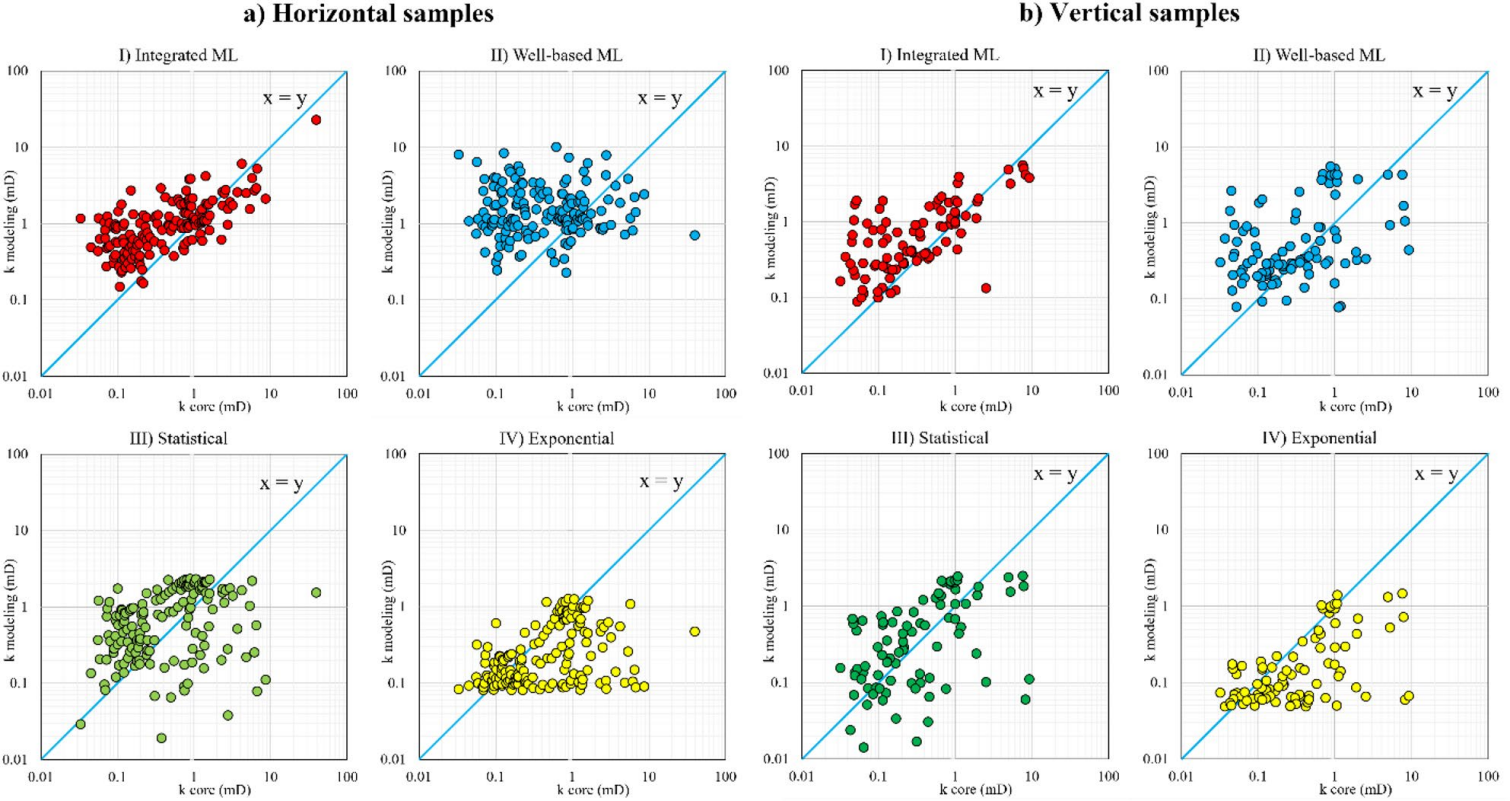
Modelling permeability versus core permeability for (**a**) horizontal core samples and (**b**) vertical core samples in the well 4; (I) integrated ML, (II) well-based ML, (III) statistical and (IV) exponential fitting models.

After constructing the fitting-based and learning-based models, in this section the modelling results are compared with laboratory core permeability data to evaluate the relative performance of the models. Firstly, the core data were fed into the models to reproduce the core permeability data. The model results were plotted against core permeability values in a log–log plot (to present a complete view of the range of the permeability data). The horizontal and vertical permeability predictions for well 2 and well 4 are illustrated in Figs. 9 and 10, respectively. The more closely the data is aligned with the unit slope line ($y=x$), the more accurate the permeability prediction is.

The data of well 2 in Fig. 9 reveals that the integrated ML method has considerably improved the permeability prediction compared with other modelling approaches. The results of the integrated ML model align with the unit slope line for both horizontal and vertical samples of the well 2. However, the well-based ML predictions using both statistical and exponential fitting methods are relatively scattered around the unit slope line and do not demonstrate a considerable progress in permeability modelling. Although the integrated ML model is working better than the other methods, it should be pointed out here that it showed a weak performance in predicting samples with a permeability of less than 0.8 mD and was mainly overestimating the permeability in this interval.

The quality of permeability predictions in the well 4 is generally lower than in the well 2 because of high uncertainty in the well 4 porosity data as described in “Results and discussion” section. Considering Fig. 10, the integrated ML delivers better predictions from the both horizontal and vertical core permeability data. Moreover, these observations demonstrate that the integrated ML alleviated the destructive effects of the low quality porosity data on vertical permeability modelling; which, led to considerable improvements in permeability prediction. The learning-based methods intelligently take advantage of the physical characteristics of the porous media by using the log parameters reducing the effect of any possible low quality data. This is the most prominent feature of applying ML models compared to the fitting-based methods that only use porosity for permeability predictions.

As shown in Figs. 9 and 10, if the deviation is above the unit slope line, the corresponding model overestimates the permeability. If it deviates under the unit slope line, then the model underestimates the permeability of the core. A comparison between the statistical and exponential models revealed that the statistical methods substantially decrease the permeability underestimation of the exponential models. According to Delfiner^21^, the permeability underestimation by exponential fitting reduces the average permeability value in 3D reservoir models.

Since the graphical representations (Figs. 9 and 10) do not sharply clarify the prominence of the integrated ML, especially for the well 4 and vertical samples, the error values were calculated by using standard metrics to present a comprehensive comparison. Mean Squared Error (MSE), Root Mean Squared Error (RMSE), and Mean Absolute Error (MAE) are the three widely used error evaluation metrics in ML and statistical analysis. They were calculated and reported in Table 5 for each modelling method by zone, well number, and permeability direction. The most efficient models (with the lowest error) were highlighted in bold in Table 5. Moreover, it should be noted that since the oil-bearing layers of the reservoir are the zones 4 and 6, the permeability modelling is only performed for these layers (for future reservoir simulations).

**Table 5:** MSE, RMSE, and MAE error values for all permeability modelling methods in each zone for the wells 2 and 4.

Well	Applied method	Zone	Horizontal samples			Vertical samples			Zone	Horizontal samples			Vertical samples		
			MSE	RMSE	MAE	MSE	RMSE	MAE		MSE	RMSE	MAE	MSE	RMSE	MAE
Well 2	Integrated ML	Zone 4	**15.2**	**3.9**	**1.9**	**24.4**	**4.9**	**2.5**	Zone 5	**12.1**	**3.5**	**1.3**	**2.2**	**1.5**	**0.7**
	Well-based ML		40.2	6.3	3.7	66.2	8.1	4.8		27.1	5.2	1.7	6.1	2.5	1.6
	Statistical		37.2	6.1	3.0	58.8	7.7	3.9		26.2	5.1	1.5	4.9	2.2	0.8
	Exponential		40.6	6.4	3.0	64.5	8.0	3.9		27.5	5.2	1.5	4.9	2.2	0.8
	Integrated ML	Zone 6	**12.3**	**3.5**	**1.6**	**21.6**	**4.7**	**2.6**	Total	**13.6**	**3.7**	**1.7**	**20.2**	**4.5**	**2.3**
	Well-based ML		34.4	5.8	2.8	54.6	7.4	3.1		35.3	5.9	2.9	53.7	7.3	3.8
	Statistical		22.8	4.8	2.1	46.8	6.8	3.1		30.5	5.5	2.4	47.1	6.9	3.2
	Exponential		24.2	4.9	2.1	48.1	6.9	3.0		32.8	5.7	2.3	50.7	7.1	3.1
Well 4	Integrated ML	Zone 4	**7.3**	**2.7**	**1.2**	0.34	**0.58**	0.34	Zone 5	–	–	–	–	–	–
	Well-based ML		31.8	5.6	2.1	0.49	0.70	0.43							
	Statistical		29.3	5.4	1.6	**0.33**	**0.58**	**0.33**							
	Exponential		31.3	5.6	1.7	0.41	0.64	0.35							
	Integrated ML	Zone 6	**0.8**	**0.9**	**0.6**	**1.54**	**1.23**	**0.89**	Total	**2.9**	**1.7**	**0.8**	**0.74**	**0.86**	**0.52**
	Well-based ML		1.2	1.1	0.7	8.97	2.99	2.47		11.8	3.4	1.1	3.32	1.82	1.11
	Statistical		1.4	1.2	1.1	4.96	2.23	1.62		11.0	3.3	1.1	1.88	1.37	0.76
	Exponential		1.1	1.1	**0.6**	6.40	2.53	1.31		11.5	3.4	0.9	2.41	1.55	0.67

In the well 2, the integrated ML shows the best performance in permeability estimation. It predicts both horizontal and vertical permeability with considerably lower error values such that the MSE of integrated ML is lower than other methods by a factor of 2–3 in all zones and directions. For samples of the well 4, the integrated ML provided the minimum error for horizontal samples in the zone 4. While, the statistical model predicts the vertical samples better in this zone. In the zone 6, both horizontal and vertical samples are well predicted by the integrated ML. Overall evaluation reveals that the integrated ML method is the most accurate, robust, and reliable permeability estimator.

**Figure 11:**
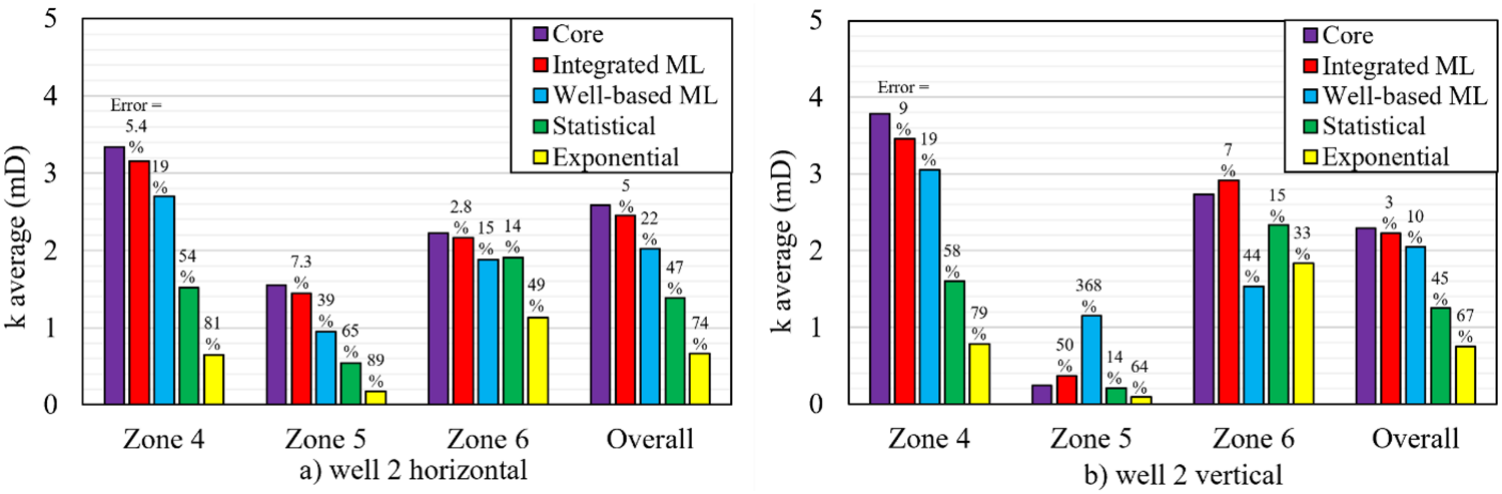
Average permeability of core samples and average of predicted permeability by models (the integrated ML, the well-based ML, the statistical and the exponential fitting) for (**a**) horizontal samples and (**b**) vertical samples in the well 2.

**Figure 12:**
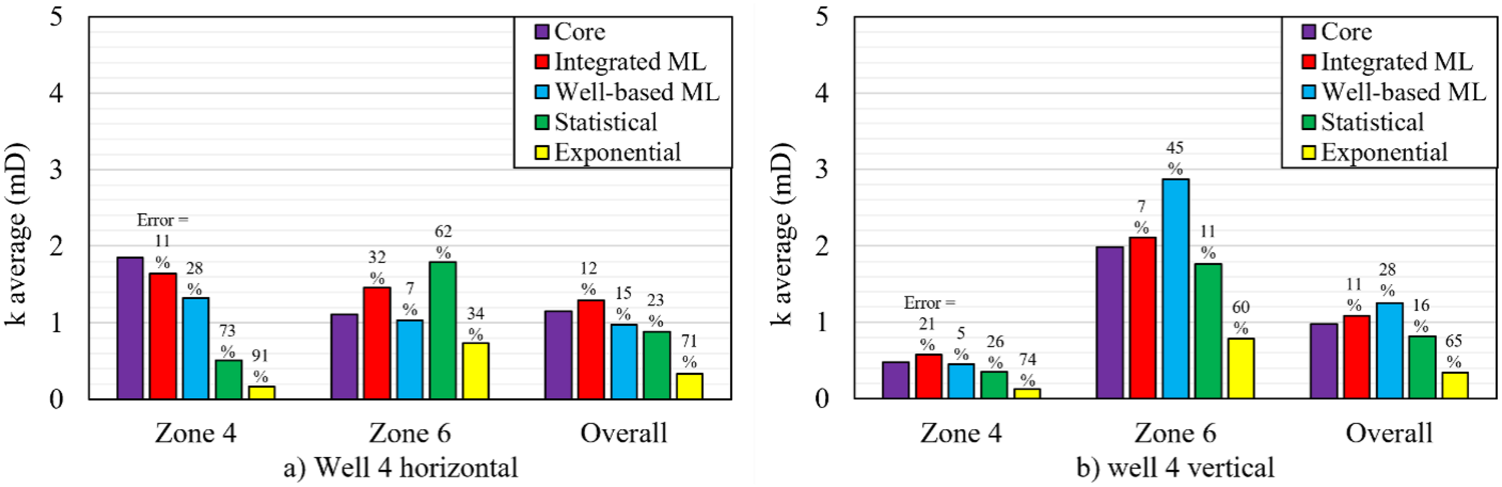
Average permeability of core samples and average of predicted permeability by models (the integrated ML, the well-based ML, the statistical and the exponential fitting methods for (**a**) horizontal samples and (**b**) vertical samples in well 4.

Another indication of the modelling performance is the ability of a modelling approach to reproduce average core permeability in each zone. This feature can guarantee that the 3D reservoir permeability model does not deviate far from the average core permeability in the zones 4 and 6. In Figs. 11 and 12, the average horizontal and vertical permeability of core samples and their corresponding model predictions are plotted for each zone of the wells 2 and 4, respectively. The error percentage (indicated above each bar) indicates the deviation of the average modelling permeability from the average core permeability (*Error* =$({k}_{core}^{avg}- {k}_{model}^{avg})/{k}_{core}^{avg}$  × 100).

In the well 2 (see Fig. 11), the integrated ML efficiently reproduced the average permeability of core samples with error values of less than 10%. For the horizontal samples of the well 4 (Fig. 12), the integrated ML method presented a suitable prediction in the zone 4. While, the well-based ML was more accurate in the zone 6. In the vertical samples of the well 4, both of the well-based and the integrated ML methods showed high efficiencies in reproducing the average permeability of core samples in the zones 4 and 6. However, overall evaluations again confirmed that the integrated ML method is the most promising predictor for average permeability prediction.

Eventually, the most efficient and promising model is the one that indicates the highest performance in four aspects: (i) having the lowest deviation from the unit slope line in Figs. 9 and 10, (ii) having the least MSE and MAE error values in Table 5, (iii) reducing the unfavorable effects of corrupt porosity data of the well 4 on permeability predictions, (iv) the minimum difference between the average core permeability and the average modelling permeability (denoted in Figs. 11 and 12). These indicators generally guarantee that the integrated ML provides the most accurate and reliable permeability predictions for this reservoir.

**Figure 13:**
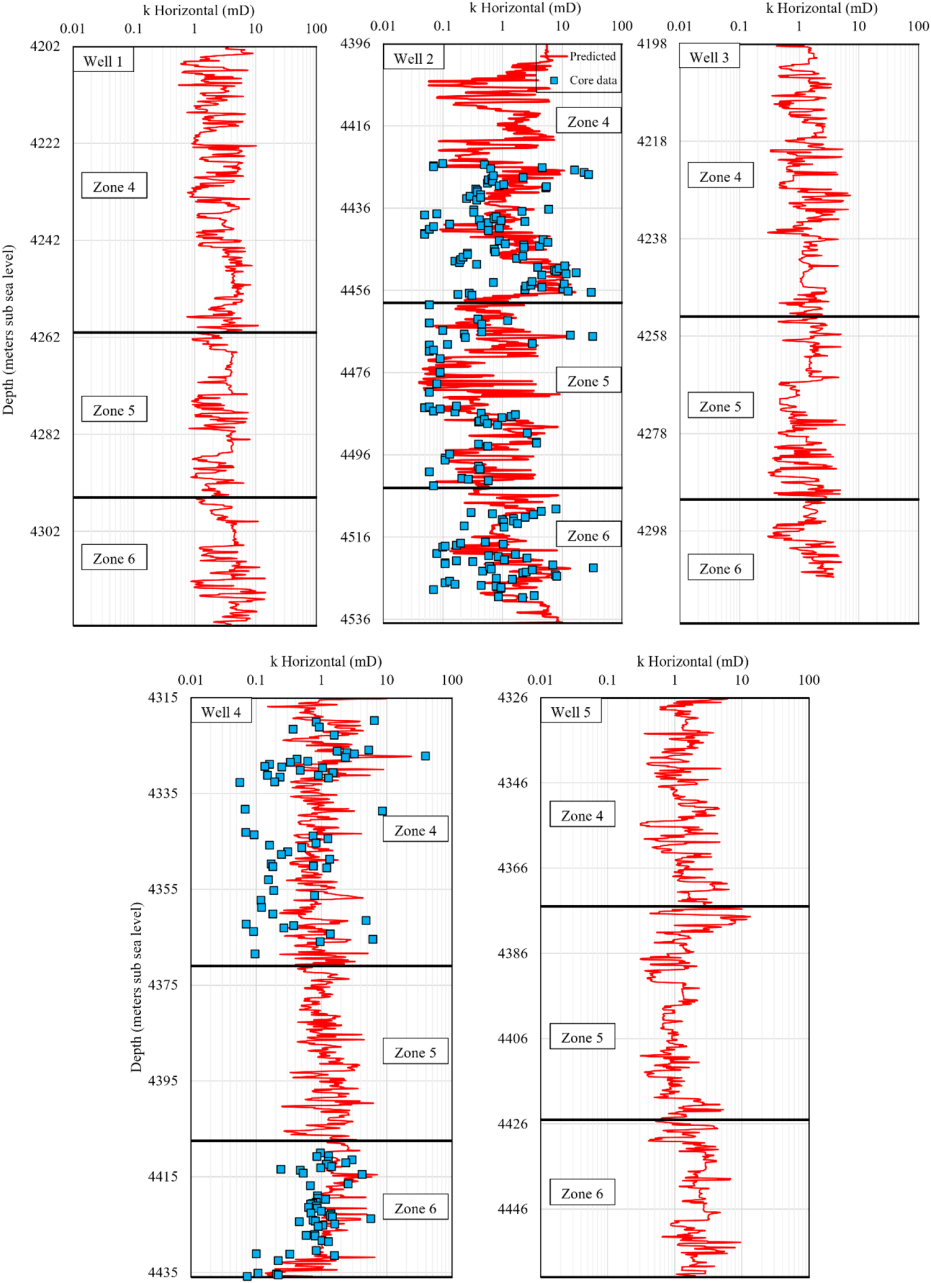
Results of horizontal permeability prediction by the integrated ML method for the zones 4, 5 and 6 in the wells 1 to 5.

**Figure 14:**
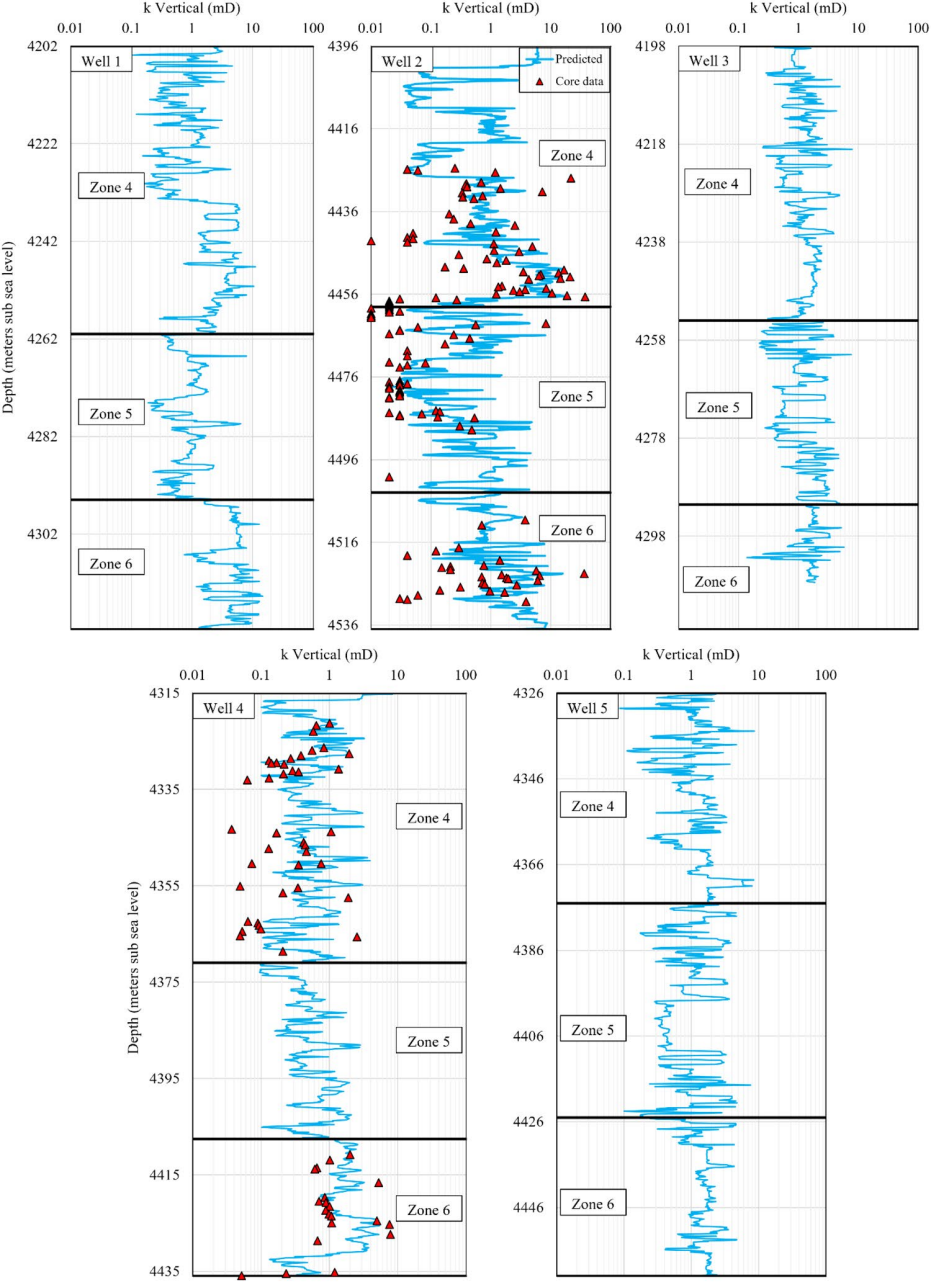
Results of vertical permeability prediction by the integrated ML method for the zones 4, 5 and 6 in the wells 1 to 5.

Since this research work is part of an extensive process of 3D reservoir model preparation for later dynamic reservoir simulations, the selected permeability modelling approach must be used to generate a 3D permeability model. The integrated ML method presented here works based on log parameters, which are available for all of the wells (1 to 5). Hence, this model provides a suitable areal and vertical data distribution throughout the 3D structure of the reservoir. After the petrophysical log data of the wells 1 to 5 were imported into the permeability model, the permeability logs were constructed for each well (Figs. 13 and 14). It can be observed that the modelling data suitably matched the core permeability data, as demonstrated in previous discussions, both graphically and numerically. The resultant permeability logs will be used to distribute the horizontal and vertical permeability in the 3D model for future dynamic reservoir simulations.

## Conclusions

In this research work, we reported one of the insignificant applications of ML methods in the field of reservoir property characterization which is vital for building 3D dynamic model of reservoirs. In the cases that enough routine and specific core data are available, it is recommended to perform adopt the following workflow; (i) to perform rock typing to identify rock types, (ii) to apply classification ML methods to predict rock type (and permeability correlation) of each point in the wells having log data. The following main points were concluded from this research work:

- A comparison between results of statistical and exponential (fitting-based) models revealed that the permeability underestimations of exponential methods were mainly alleviated using statistical methods.
- In highly heterogeneous, low porosity/permeability, and tight oil reservoirs (which is the case in this research work), the standard fitting-based methods failed to model reservoir rock permeability efficiently, precisely. Hence, the ML permeability modelling approach demonstrated to be more robust and accurate in such cases.
- Considering the limited data and the low areal distribution of the wells in this reservoir, the permeability modelling using log data (instead of core data) included a better vertical and areal data distribution of the reservoir structure into the permeability modelling process.
- Using the log data improved the reservoir permeability model because it used more physics of the reservoir rock, such as sonic characteristics, radioactivity, and electric features, instead of only using porosity correlations (as in fitting-based methods).
- Multiple graphical and quantitative evaluations demonstrated that the integrated ML model is considerably more efficient than the well-based ML, exponential, and statistical methods. Best predictions made by the integrated ML (i.e., RF model) mainly owing to its database, which is more extensive than the database used in well-based ML. According to the results obtained from the integrated model, the RMSE for horizontal permeability in well 4 and well 2 are 1.7 and 3.7 respectively, while for vertical permeability, they are 0.86 and 4.5 respectively.
- Proper application of ML methods and log data for permeability prediction significantly reduced undesirable effects of discrepancy and/or uncertainty in the dataset.

As a result of this research work, permeability modelling should incorporate learning-based approaches and techniques to better address challenges such as data gaps, low quality, scattering, potential errors, and discrepancies in data collection. By reducing the impact of these issues on outputs, these methods increase reliability and precision. Incorporating these intelligent procedures will significantly enhance future research initiatives and operational approaches in similar geological settings.

## Data Availability

The data supporting this research work are available from the corresponding authors upon reasonable request and with the permission of the IRD.

## Abbreviations

AI: Artificial intelligence
ANFIS: Artificial neuro-fuzzy interference system
ANN: Artificial neural network
BS: Bit size
CALI: Caliper
CMEF: Committed machine with empirical formulas
CT: Total conductivity
DEN: Formation density log
DRT: Discrete rock typing
DT: Decision tree
DT: Sonic log
EOR: Enhanced oil recovery
F: Formation factor
FFBP: Feed-forward back-propagation
FL: Fuzzy logic
FZI: Flow zone indicator
GA: Genetic algorithm
GBR: Gradient boosting regressor
GMDH: Group method of data handling
GR: Gamma ray
HGAPSO: Hybrid GA and PSO
ICA: Imperialist competitive algorithm
KNN: K-nearest neighbors
LLD: Deep laterolog
LLS: Shallow laterolog
LM: Levenberg–Marquardt
LSSVM: Least-square support vector machine
MAE: Mean absolute error
MIMO: Multiple-input multiple-output
MKF: Mixed kernel function
ML: Machine learning
MSE: Mean squared error
MSFL: Micro spherically focused log
NMR: Nuclear magnetic resonance
NPHI: Neutron-porosity log
PEF: Photoelastic adsorption factor
PHID: Density porosity
PHIE: Effective porosity
PHIT: Total porosity
R: Correlation coefficient
R^2^: Coefficient of determination
RBF: Radial basis function
RD: Deep resistivity
RF: Random forest
RHOB: Deep density log
RHOZ: Formation density
RL: Resistivity log
RLA1: Apparent resistivity focusing mode 1
RLA5: Apparent resistivity focusing mode 5
RMSE: Root mean squared error
RQI: Rock quality index
RT: Total resistivity
RT: Electrical resistivity
RTs: Rock types
RVR: Relevance vector regression
SFL: Spherically focused log
SGR: Spectral Gamma Ray
SRT: Static rock typing
SVM: Support vector machine
SWT: Water saturation
TNPH: Thermal neutron porosity well logs
VOL_CALCITE: Volumes of calcite
VOL_DOLOM: Volumes of dolomite
VOL_UOIL: Volumes of oil
VOL_UWATER: Volumes of water
VSH: Shale volume
XGBoost: Extreme gradient boosting
